# State Microscopical Society

**Published:** 1879-06

**Authors:** 


### ARTICLE X.

### STATE MICROSCOPICAL SOCIETY.

At the annual meeting of the State Microscopical Society, held at the Academy of Sciences, 263 Wabash Av., April 25th, the following officers were elected for the coming year:

President, H. A. Johnson, M.D.; Vice President, Prof. H. H. Babcock; Vice President, Lester Curtis, M.D.; Treasurer, W. H. Summers; Recording Secretary, Ernest Stuart; Corresponding Secretary, Wm. T. Belfield, M.D.; Trustees, H. W. Fuller, Prof. E. S. Bastin, Jas. Colgrove, B. W. Thomas and H. F. Atwood.

The following is an abstract of a paper on "Tactile Hairs" read before the State Microscopical Society:

### THE TACTILE HAIRS COMMONLY CALLED WHISKERS, OF CERTAIN ANIMALS.

By DR. LESTER CURTIS, of Chicago.

The growth of an ordinary hair takes place by the multiplication of the cells which surround the papilla and the follicle possesses no muscles of volition or special arrangement of nerve fibers.

The first noticable peculiarity of the tactile hair, as in the snout of a mouse, is the great size of the follicle, several times that of an ordinary hair follicle; this follicle has a bulging about one-third of the distance from the top which corresponds with a remarkable enlargement within.

Two-thirds of the lower portion of the follicle is surrounded by striated muscular fibers which appear to arise from the side of the follicle by a short tendon. They encircle the follicle and then pass off to a considerable distance to become lost in the muscular fibers of the lip. These muscles are furnished with a rich plexus of unusually large capillaries. Other muscles are attached to the hair in a manner somewhat similar to the ordinary erectors of the hair, but are more abundant and of the striated variety.

Within the follicle and outside the root sheath of the hair is close plexus of very fine capillaries which send branches over the enlargement mentioned above and form a peculiar ring-like plexus above it. Between the branches of this ring-like plexus there is but little tissue; but in the mouse and cat, at least, it does not form a sinus as is claimed by some.

The enlargement surrounds the hair eccentrically and consists of two parts, the outer of which when the follicle is divided longitudinally through the center of the hair, is nearly circular in outline and is attached to the inner part by a short, thick pedicle. It is interspersed with oblong nuclei. The inner part is not so wide but is longer and surrounds the root-sheath like a band.

The speaker considered these last organs to be of a nature similar to the terminal organs of nerves found in other portions of the body but was not prepared at present to demonstrate his position.

The paper closed with a brief biblography of the subject, after which numerous slides were shown in illustration of his descriptions of these organs.

